# Cytokines Levels and Salivary Microbiome Play A Potential Role in Oral Lichen Planus Diagnosis

**DOI:** 10.1038/s41598-019-54615-y

**Published:** 2019-12-02

**Authors:** Maria Fernanda Marques Silva de Carvalho, Denise Cavalieri, Sabrina Do Nascimento, Talita Gomes Baeta Lourenço, Danielle Viana Ribeiro Ramos, Denise da Cunha Pasqualin, Leandro Aurélio Liporoni Martins, Fernanda Agostini Rocha, Débora Heller, Luciana Marti

**Affiliations:** 1 Hospital Israelita Albert Einstein, Experimental Research, São Paulo, Brazil; 2 Secretaria Municipal de Saúde de São Paulo, Centro de Especialidades Odontológicas III Alto da Boa Vista, São Paulo, Brazil; 3 Universidade Federal do Rio de Janeiro, Oral Microbiology Laboratory, Rio de Janeiro, Brazil; 4 Hospital Albert Einstein - Instituto Israelita de Responsabilidade Social, São Paulo, Brazil; 5 Hospital Israelita Albert Einstein - Laboratório de Anatomia Patológica, São Paulo, Brazil; 6 Universidade Cruzeiro do Sul - College of Dentistry, São Paulo, Brazil

**Keywords:** Interleukins, Mucosal immunology

## Abstract

Oral lichen planus (OLP) is a chronic Th1-mediated inflammatory mucocutaneous disease of the skin and oral mucosa that can have various clinical presentations. Lesions are usually bilateral and often painful. While cutaneous Lichen Planus (LP) lesions are self-limiting, the oral lesions are chronic and rarely remissive. The diagnosis of oral lichen planus (OLP) is often challenging, and confirmation by histopathological criterion is generally advised. The aim of our study was to identify the cytokines present in OLP-suggestive lesions and in non-specific inflammatory lesions (NSIL) used as controls. Moreover, assess cytokines protein levels and oral microbiota composition in whole saliva samples. Histopathological analysis, immunohistochemistry and gene expression were used as techniques to analyze the oral mucosal tissue samples. ELISA was conducted to analyze salivary cytokine levels and 16S rRNA sequencing was used to determine the salivary microbiome. As a result we observed larger number of infiltrated lymphocytes (p = 0.025), as well, more T CD4 lymphocytes in the epithelial tissue (p = 0.006) in OLP samples compared to NSIL. In addition, the OLP samples displayed more apoptotic cells compared to NSIL (p = 0.047). Regarding the cytokine analysis, IFN-γ and IL-33 were more expressed in OLP lesions than in NSIL samples (p < 0.001; p = 0.026). Furthermore, our results demonstrated higher levels of IFN-γ protein expression in the saliva of OLP group compared to controls (p = 0.0156). We also observed noted differences in the oral microbiota composition between OLP and NSIL saliva samples. In conclusion, OLP lesions presented larger numbers of apoptotic and inflammatory cells, higher levels of IFN-γ and IL-33 compared to NSIL, and these lesions also differ regarding oral microbiota composition. These results are consistent with the Th-1-mediated chronic inflammation nature of oral lichen planus investigated lesions and displayed unique features that could be used as a diagnostic tool.

## Introduction

Oral lichen planus (OLP) is a chronic and inflammatory mucocutaneous disease of the skin and oral mucosa that can have various clinical features. OLP affects mainly 40 to 70 years old persons being more prevalent in women. OLP lesions inside the oral cavity rarely undergo spontaneous remission whereas cutaneous lichen planus lesions are self-limiting. Correctly diagnosing OLP is often challenging, hence, it is worth to emphasize that it should always be guided by clinical and histopathological criteria, because OLP lesions subtypes may become malignant^1–3^. The main histopathological features of this disease are apoptosis of basal keratinocytes and basement membrane disruption together with a dense sub epithelial lymphocyte infiltrate^3^. The etiology of OLP remains unclear; however, there are suggestive results that OLP may be an immune-mediated process led by CD4 T cells and possibly related to unspecific inflammation and/or infection. Numerous findings have supported the idea that immune imbalances and complex cytokine networks may play important roles in the OLP chronic profile^4^. For instance, a high proportion of the novel CD4+ T helper cells subset-Th17 cells were detected in OLP lesions. These cells would release inflammatory cytokines leading to migration and activation of inflammatory cells and cytotoxic CD8 T cells with consequent damage to basal keratinocytes and their death by apoptosis^5–10^. In specific cytokine environments, CD4 T cells can differentiate into various subsets, such as Th1, Th2, Th9, Treg and Th17 cells^10^. Interleukin 2 (IL-2) and interferon-gamma (IFN-γ) are the cytokines that drive predominantly Th1 differentiation and IFN-γ was shown to be an important inflammatory cytokine present in OLP lesions^5,6,11^. In addition, Th17 cells have also been identified in OLP lesions^12^ and the authors correlated the larger number of Th17 cells with the erosive type of OLP lesions rather than with the reticular type^13^. Interleukin 33 (IL-33) is a cytokine that can drive strong Th2 responses, but can also help Th1 immune responses and activate CD8 T cells^14^. Recently, the presence of IL-33 was also reported in OLP lesions^15^.

On the other hand, infections with oral microorganisms have been described and several viruses were related to OLP^16,17^. In addition, there are studies that have correlated *Candida albicans* infection with several OLP cases^18^. Others studies have correlated *Helicobacter pylori* with OLP, but there are divergences between different sources^19,20^. Even though, these events are not directly related to the OLP etiology, all these factors might influence the oral microbiome composition and as consequence alter the local immunity and thus favoring the OLP maintenance and progression.

Given the incipient knowledge on OLP etiology and the cytokines participation in OLP, the aim of the present study was to evaluate the gene expression of IFN-γ, IL-17 and IL-33 cytokines, oral microbiome composition and immunohistochemistry analyses with histopathological profile of oral mucosal tissue samples from OLP lesions in order to better diagnosis this disease. Non-specific inflammatory lesions (NSIL) of the oral mucosa were analyzed as controls.

## Materials and Methods

### Ethics

Patients who presented lesions in the oral mucosa with clinical features suggestive of oral lichen planus (OLP), and agreed to participate, were included in the study after signing a written informed consent form. This work was conduct according to Helsinki declaration. The project was previously approved by the ethics committees of the Hospital Israelita Albert Einstein and of the São Paulo Municipal Health Division – Secretaria Municipal da Saúde - SMS-SP under CAAE number: 55053716.5.3001.0086. In addition, all methods were performed in accordance with the relevant guidelines and regulations and the original datasets generated and/or analyzed during the current study are available from the corresponding author on reasonable request.

### Acquisition of samples

The tissue samples were collected from the lesions during routine diagnosis confirmatory biopsies from patients attended at the Center of Dental Specialties (CEO), Southern Regional of the City of São Paulo. A total of 15 tissue samples were obtained from patients with clinically white oral mucosal lesions. Each tissue sample was halved; one half was immediately stored in 10% formaldehyde solution and sent to histological diagnosis and the other half was immediately frozen in liquid nitrogen for preservation and sequential gene expression analysis. After histological analysis, six samples were diagnosed as OLP and nine samples were diagnosed as non-specific inflammatory lesions (NSIL) and considered as control samples.

Unstimulated whole saliva samples (uWS) were also collected from the patients before the biopsy procedure. These samples were collected using a system for collecting oral cavity fluids^21^ according to the manufacturer’s instructions (SuperSAL Collection Device, Oasis Diagnostic Corporation), and a total volume of approximately 2 ml of uWS was obtained from each patient. The duration of the collection was recorded, and salivary flow rate was calculated. The samples were kept on ice throughout the collection period and during transport to the laboratory for processing^22^. After, the tubes containing saliva samples were centrifuged (Eppendorff, 5430 R) at 14000 g for 20 min at 4 °C, next the supernatant was collected, aliquoted and stored at −80 °C until the analysis. The uWS sediments were also stored at −80 °C for future microbiome analysis.

### Total RNA extraction and cDNA synthesis

Total RNA was isolated from the tissue specimens using the RNeasy^®^ Micro kit (Qiagen) following the manufacturer´s instruction. The purified total RNA quality was assessed by spectrophotometry using the NanoVue (GE Healthcare, UK). A total volume of 1μg of the extracted RNA was reverse transcribed into cDNA using the Quantitec Reverse Transcription kit (Qiagen).

### Quantitative real time polymerase chain reaction (qRT-PCR) analysis

PCR amplifications were performed on the ABI Prism 7500 (Applied Biosystems). Gene expression assays for IL-17A (NM_002190), INF-γ (NM_0 00619) and IL-33 (NM_033439.3) were performed and analyzed.

The gene-specific primers used for amplification with QuantiFast® SYBR® Green PCR Kit; and their sequences were:

18S (RPL13a)- Forward-“TTGAGGACCTCTTGTGTATTTGTCAA” and Reverse-“CCTGGAGGAGAAGAGGAAAGAGA”;

IL-17- Forward-“ACCGGAATACCAATACCAATCC” and Reverse-“GGATATCTCTCAGGGTCTGCATTAT”;

IFN-y- Forward-“GGTCATTCAGATGTAGCGGATA” and Reverse-“AGACAATTTGGCTCTGCATTAT”;

IL-33- Forward-“GAAGAACACAGCAAGCAAAG” and Reverse-“GAAGAACACAGCAAGCAAAG”.

The amplification program consisted of initial denaturation at 95 °C for 1 minute followed by 40 cycles at 95 °C for 10 seconds, and an annealing and extension phase at 60 °C for 30 seconds. The qRT-PCR assays were performed in technical triplicates to minimize the effects of unequal quantities of starting RNA and to eliminate potential sources of inconsistency. Relative expression levels of each gene were normalized to ribosomal protein (RPL13a) using the 2^-ΔΔ^ Ct method.

### Histopathological analysis

Formalin-fixed paraffin-embedded OLP and NSIL samples were sectioned into 3 µm thick slices and transferred to microscope slides. The tissue was stained with hematoxylin–eosin (HE), and these slices images were performed using Aperio ScanScope microscopy (Leica Biosystem, Germany) for images scanning. The analysis of the tissue sections was performed by two expert pathologists from the Clinical Pathology Laboratory of Albert Einstein Hospital.

### Elisa assay

The concentration of IFN-γ in the supernatant of Whole Saliva Samples (WSS) was determined using ELISA kit (XpressBio Life Science Products, Frederick, MD, USA), according to the manufacturer’s instructions. The saliva samples were diluted 1:10 and 1:5. Briefly, anti-IFN-γ antibody was pre-coated into 96-well plates and biotin conjugated anti-IFN-γ antibody was used as detection antibodies. The standards, test samples and biotin conjugated detection antibody were added to the wells subsequently. HRP-Streptavidin was added, and unbound conjugates were washed away with wash buffer. TMB substrates were used to visualize HRP enzymatic reaction. The final plate reading was performed using the 450 nm O.D. absorbance in the microplate reader DTX 880 Multimode Detector (Beckman Coulter, Brea, CA). The concentration (pg/mL) of IFN-γ in the saliva samples was calculated using the values determined by the standard curve.

### Immunohistochemistry

Immunohistochemistry staining for CD3, CD4 and CD8 T lymphocytes was performed on OLP and NSIL paraffin-embedded formalin-fixed tissue sections using the EnVision Flex Kit (Dako) according to the manufacturer’s instructions. All tests were performed on the Autostainerlink 48 (Dako) equipment. Polyclonal CD3, CD4 (clone: 4B12) and CD8 (clone: C8/144) antibodies from DAKO were used for the labeling. These data were analyzed by a Pathologist from the Clinical Pathology Laboratory of Albert Einstein Hospital. The absolute number of cells was obtained by counting cells per area.

### Salivary microbiome sequencing and data processing

The data presented in this manuscript was generated by Neoprospecta Microbiome Technologies as previously described^23,24^. Briefly, microbial DNA from four (2 OLP and 2 NSIL) saliva pellet samples was obtained using AMPureXP beads (Beckman Coulter, Brea, CA) after a thermal lysis process of 96 °C for 10 min. Amplicon sequencing library preparation was performed for bacteria using the V3–V4 16S rRNA gene primers 341F (CCTACGGGRSGCAGCAG, 10.1371/journal.pone.0007401) and 806R (GGACTACHVGGGTWTCTAAT, 10.1038/ismej.2012.8), with the following conditions: the first PCR primers contain the Illumina sequences based on TruSeq structure adapter (Illumina, San Diego, CA), allowing the second PCR with indexing sequences. The PCR reactions were always carried out in triplicates using Platinum Taq (Invitrogen, USA). The library pool was adjusted to a final concentration of 11.5 pM and sequenced in a MiSeq system, using the standard Illumina primers provided in the kit. A single-end 300 nt run was performed using a V2x300 sequencing kit. After sequencing, the bioinformatics pipeline performs sequence demultiplexing, adaptor and primer trimming. The reads were size normalized to 283pb. Read quality filter (E) was performed converting each nucleotide Q score in error probability (ei), clustered sequences (OTUs) were subjected to taxonomic classification comparing them with a 16S rRNA database (NeoRefdb, Neoprospecta Microbiome Technologies, Brazil). Sequences with at least 99% of identity in the reference database were taxonomically assigned and the samples were then evaluated based on their microbiome composition, focusing on bacterial profiles and their quantity amounts. A rarefaction stage was performed before diversity analysis, standardizing the samples for a total of 10,000 sequences per sample, using QIIME pipeline version 1.9.124. The alpha diversity was calculated using the Shannon indices^25^ and compared between groups using nonparametric two sample t-tests, and the default Monte Carlo permutations. We also ran random forest analysis using the out-of-bag method of prediction error, carried out to classify the clinical status of individuals based on the number of reads of different OTUs. The mean decrease in accuracy was assessed for each OTU to determine the variables of importance for prediction by removing the association between that variable and the target (clinical status). This was achieved by randomly permuting the values of the variable and measuring the resulting increase in error.

### Statistic data analysis

Numeric data are shown as means accompanied by standard deviation (SD), or by medians and quartiles accompanied by minimum and maximum values. Categorical variables are shown as absolute and relative frequencies. Fisher’s exact test was performed to verify associations between histopathological diagnosis and categorical data of patients and lesions. Student’s t-test was performed to compare patient age between OLP and NSIL groups; the Mann-Whitney test was used to analyze cytokine expression levels. The statistical package SPSS (SPSS Statistics for Windows, Version 24.0, Armonk, NY: IBM Corp, 2016) was used for the analyses and the significance levels were set at 5% (*p* value of 0.05).

## Results

### Demographic and clinical data of patients

The demographic and clinical data of patients included in this study are shown in Table 1 and comprise gender, age, smoking habit, symptoms, use of medication, use of dental prosthesis, and presence of systemic diseases such as hypertension and diabetes. The mean uWS flow rate was 0.33 ± 0.18 ml/min (range: 0.2–0.7 ml/min). After data analysis, no significant differences relative to these aspects were found between OLP and NSIL groups.

**Table 1:** Demographic data of patients involved in this study.

	Total of patients (n = 15)	Histological diagnosis		*p* value
		NSIL (n = 9)	OLP (n = 6)	
Gender				0,622^FT^
Female	6 (40,0%)*	3 (33,3%)	3 (50,0%)	
Male	9 (60,0%)	6 (66,7%)	3 (50,0%)	
Age				0,498^TT^
Mean (SD)	66,6 (7,0)	65,6 (8,7)	68,2 (3,0)	
Minimum; Maximum	56; 81	56; 81	66; 72	
Smoking habit				>0,999^TF^
Non-smoking	7 (46,7%)	4 (44,4%)	3 (50,0%)	
Smoking	8 (53,3%)	5 (55,6%)	3 (50,0%)	
Symptoms				0,089^TF^
No	10 (66,7%)	8 (88,9%)	2 (33,3%)	
Yes	5 (33,3%)	1 (11,1%)	4 (66,7%)	
Medication				>0,999^TF^
No	3 (20,0%)	2 (22,2%)	1 (16,7%)	
Yes	12 (80,0%)	7 (77,8%)	5 (83,3%)	
Dental Prosthesis				0,329^TF^
No	5 (33,3%)	2 (22,2%)	3 (50,0%)	
Yes	10 (66,7%)	7 (77,8%)	3 (50,0%)	
Hypertension				>0,999^TF^
No	3 (20,0%)	2 (22,2%)	1 (16,7%)	
Yes	12 (80,0%)	7 (77,8%)	5 (83,3%)	
Diabetes				0,622^TF^
No	9 (60,0%)	6 (66,7%)	3 (50,0%)	
Yes	6 (40,0%)	3 (33,3%)	3 (50,0%)	

**NSIL:** Nonspecific inflammatory lesions; **OLP:** Oral lichen planus;; **TT:** student’s t test; **FT:** Fisher’s exact test; **SD:** standard deviation. *Percentage in relation to total.

### Nature and characteristics of the inflammatory lesions in OLP and NSIL samples

Histopathological analysis showed the characteristic alteration found in OLP. Namely, dense band lymphomonocytic infiltrate in the lamina propria, disruption of the basal layer with bulla formation and consequently basal layer degeneration, vacuolar interface alterations, acantosis, hiperqueratosis, large numbers of apoptotic cells and absence of dysplasia in the tissue. In contrast, tissue samples classified as NSIL had a less dense, diffuse, irregularly distributed in the lamina propria and sometimes also presence of polymorphonuclear cells such as eosinophil, few or no disturbance or degeneration of the basal layer, no bullae formation, and low numbers or absence of apoptotic cells along the tissue.

**Figure 1:**
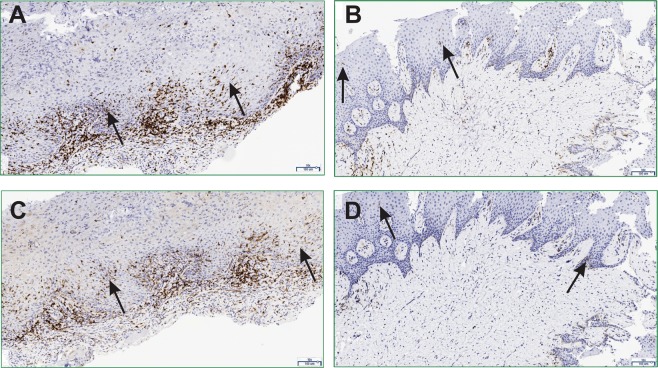
Lymphocytes infiltrating in OLP and NSIL patients samples**:** Slices of OLP (**A**,**C**) or NSIL (**B**,**D**). Human mucosal lesions samples were submitted to immunohistochemical assay to analyze the CD3 (**A**,**B**) and CD4 (**C**,**D**) intraepithelial expression. The images are representative of OLP (n = 6) and NSIL (n = 9) samples. Scar bar: 100 µm.

The immunohistochemistry staining showed a strong band-like infiltrate of CD3 lymphocytes below the basal layer in OLP lesions compared to control group (NSIL), whereas in NSIL, CD3 lymphocyte infiltrate was mild or moderate (p = 0.025) (Fig. 1A,B). In addition, CD4 cells in the epithelium were scarce (between 0–10 cells/area) in NSIL sections, while in OLP lesions they were in larger numbers (>25 cells/area) in the epithelium (p = 0.006) (Fig. 1C,D).

There was no significant association between the OLP and NSIL groups when comparing the numbers of CD4 lymphocytes infiltrating the connective tissue, CD8 lymphocytes infiltrating the epithelium (p = 0.066) or CD8 lymphocytes in the lamina propria (p = 0.126).

The presence of apoptotic cells was more conspicuous in sections from patients with OLP than in the control group and a positive correlation was found regarding this aspect of histopathology (p = 0.047). Other morphological alterations such as suprabasal disruption, vacuolar interface, acantosis, hiperqueratosis, paraqueratosis, papilomatosis and presence of melanophages were not considerably different between the groups (Table 2

**Table 2:** Biopsy samples histological features.

	Total of patients (n = 15)	Histological diagnosis		*p* value
		NSIL (n = 9)	OLP (n = 6)	
Vacuolar interface alterations				0.168^TF^
None	9 (60.0%)	7 (77.8%)	2 (33.3%)	
Incipient	1 (6.7%)	1 (11.1%)	0 (0.0%)	
Few	1 (6.7%)	0 (0.0%)	1 (16.7%)	
Present	4 (26.7%)	1 (11.1%)	3 (50.0%)	
Suprabasal Disruption				0.748^TF^
None	9 (60.0%)	6 (66.7%)	3 (50.0%)	
Discreet	1 (6.7%)	0 (0.0%)	1 (16.7%)	
Present	5 (33.3%)	3 (33.3%)	2 (33.3%)	
Apoptotic cells				^**TF**^
None	10 (66.7%)	8 (88.9%)	2 (33.3%)	
Few	4 (26.7%)	1 (11.1%)	3 (50.0%)	
Present	1 (6.7%)	0 (0.0%)	1 (16.7%)	
Hiperqueratosis				>0.999^TF^
No	3 (20.0%)	2 (22.2%)	1 (16.7%)	
Yes	12 (80.0%)	7 (77.8%)	5 (83.3%)	
Paraqueratosis				0.486^TF^
No	2 (13.3%)	2 (22.2%)	0 (0.0%)	
Yes	13 (86.7%)	7 (77.8%)	6 (100.0%)	
Papilomatosis				0.229^TF^
No	12 (80.0%)	6 (66.7%)	6 (100.0%)	
Yes	3 (20.0%)	3 (33.3%)	0 (0.0%)	
Acantosis				>0.999^TF^
Yes	15 (100.0%)	9 (100.0%)	6 (100.0%)	
Melanophages in the chorion				0.525^TF^
None	12 (80.0%)	8 (88.9%)	4 (66.7%)	
Rare	3 (20.0%)	1 (11.1%)	2 (33.3%)	

**NSIL:** Nonspecific inflammatory lesions; **OLP:** Oral lichen planus; **FT:** Fisher’s exact test.

).

### Expression levels of IL-17, IFN-γ and IL-33

OLP lesions presented significant higher gene expression of IFN-γ and IL-33 compared to the control group. Significant differences in IFN-γ (*p* < 0.001) and IL-33 (*p* = 0.026) relative gene expression levels were found in OLP lesions compared to control lesions (Table 3).

**Table 3:** Cytokines genic expression.

	Total of patients (n = 15)	Histological diagnosis		*p* value
		NSIL (n = 9)	OLP (n = 6)	
IFN-γ				**<0**.**001**^**MW**^
Median (Q1; Q3)	1.1 (1.0; 69.0)	1.0 (1.0; 1.0)	92.3 (12.1; 123.2)	
Minimum; Maximum	1.0; 3352.2	1.0; 1,2	4.5; 3352.2	
IL-33				**0**.**026**^**MW**^
Median (Q1; Q3)	1.0 (1.0; 1.8)	1,0 (1.0; 1.0)	2.1 (1.1; 4.5)	
Minimum; Maximum	0.0; 14.3	0.0; 1.0	0.0; 14.3	
IL-17				0.328^MW^
Median (Q1; Q3)	1.0 (0.0; 1.0)	1.0 (1.0; 1.0)	0.0 (0.0; 1.8)	
Minimum; Maximum	0.0; 117.7	1.0; 1.0	0.0; 117.7	

**NSIL:** Nonspecific inflammatory lesions; **OLP:** Oral lichen planus; **MW:** Mann-Whitney test; **Q1:** first quartile; **Q3:** third quartile.

On the other hand, the relative gene expression of IL-17 was not different between OLP and control lesions (*p* = 0.328) (Table 3).

### Salivary levels of IFN-γ in whole saliva samples

**Figure 2:**
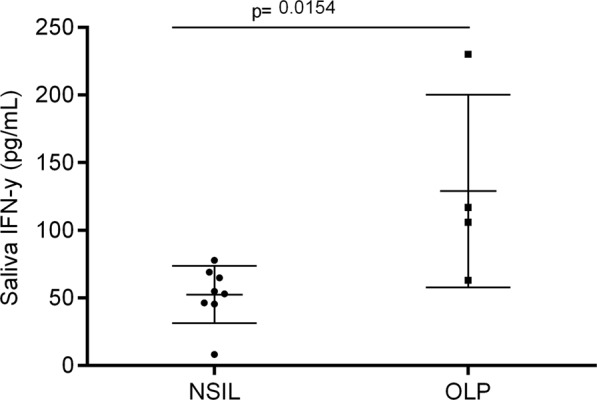
Protein expression of IFN-γ in saliva of patients with OLP or NSIL: Whole saliva samples from patients with OLP (n = 4) or NSIL (n = 8) were evaluated for the IFN-γ levels concentration (pg/mL) by ELISA assay. The measurement were considered statistically significant when p < 0.05.

The IFN-γ protein level in salivary samples were higher in OLP (140 pg/mL) compared to NSIL control samples (50 pg/mL) (*p* = 0.0156) (Fig. 2). Thus, this result correlates with the higher level of IFN-γ observed in genic expression. The OLP saliva sample that presented the lowest level of IFN-γ was the only one that expressed high levels of mRNA for IL-17.

### Salivary microbiome

**Figure 3:**
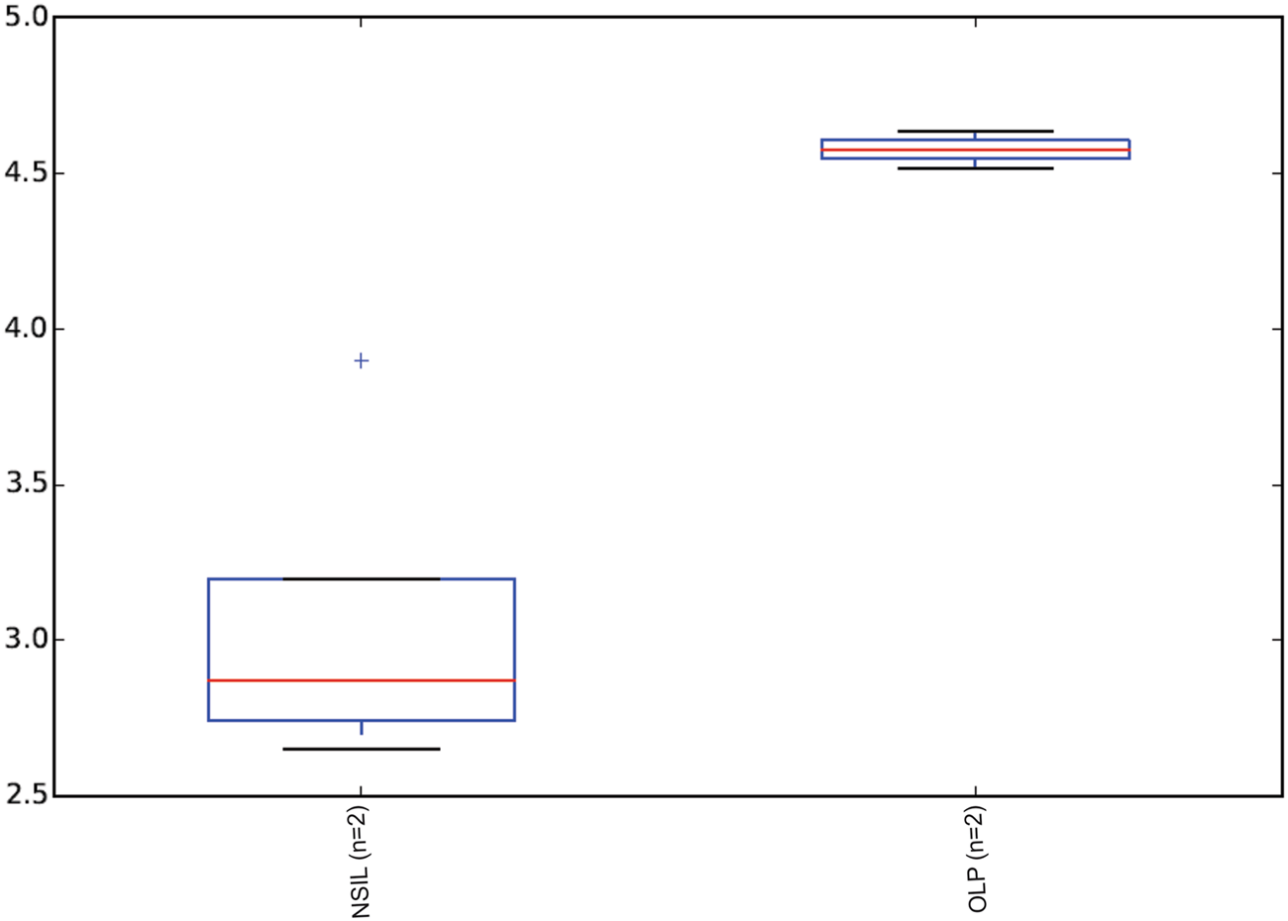
Alpha diversity. Alpha diversity using Shannon indices, calculated for each clinical group (p < 0.05, Student t test).

**Figure 4:**
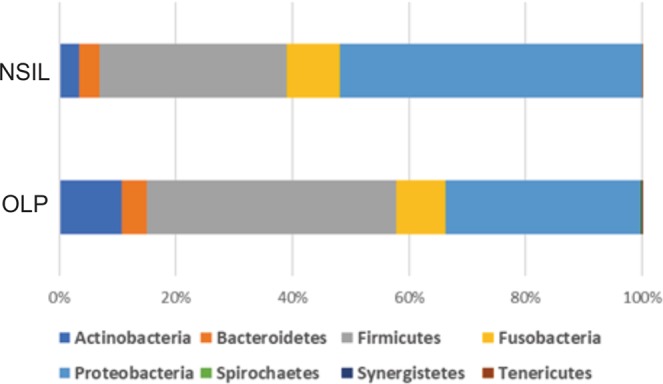
Proportional taxonomic assignments at the phylum level: Represents the saliva samples from individuals with OLP and NSIL. No significant differences among groups were observed.

**Figure 5:**
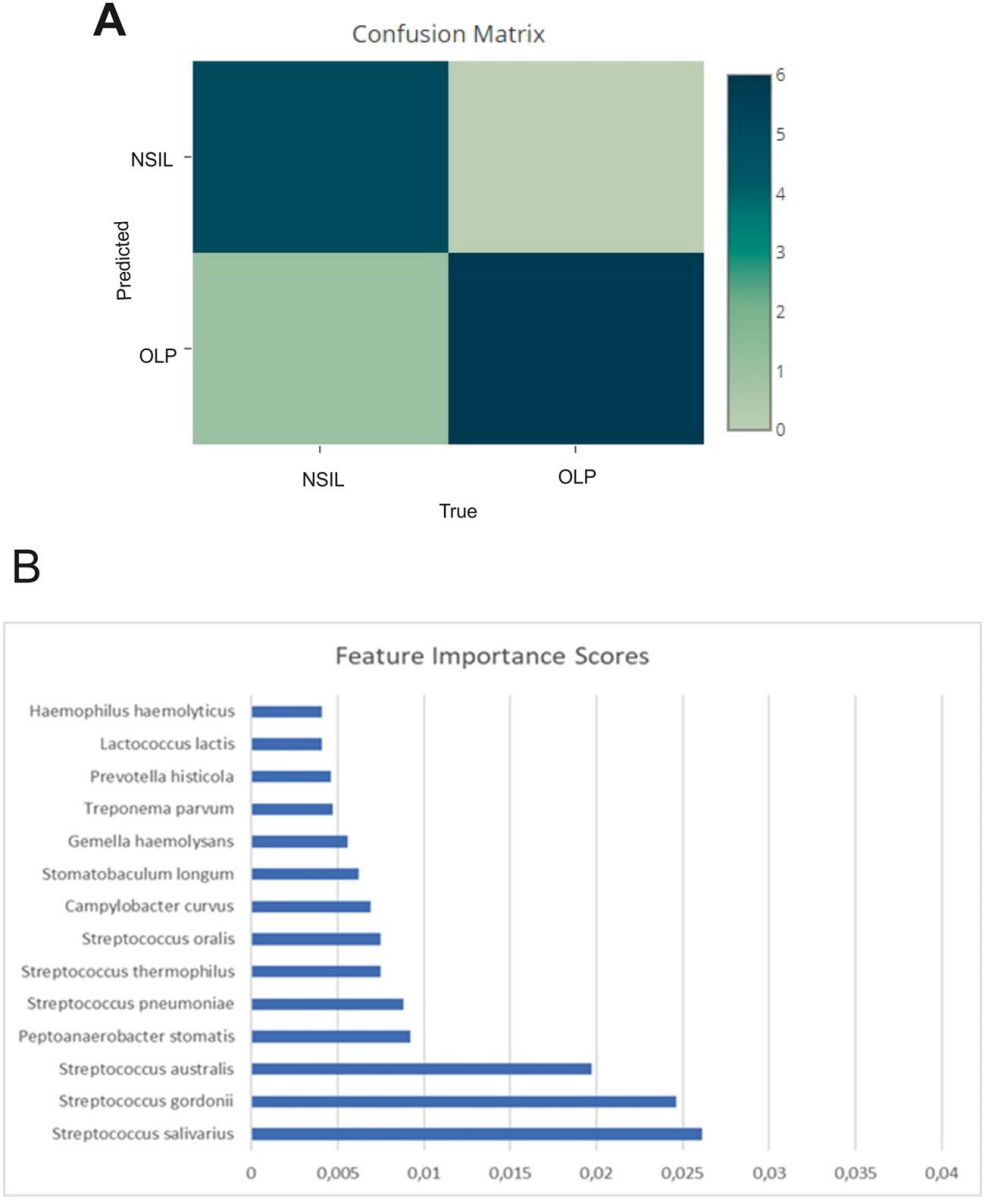
Confusion Matrix and Important variables. (**A**) Confusion Matrix based on counts of OTUs for classification of patients. Lines represent the actual number of patients in each clinical condition and the columns represent the number of patients classified from the OTUs in each clinical condition. Colors represent the number of patients as indicated in the bar on the right. (**B**) Important variables in the classification of clinical groups.

To gain insight into the salivary microbiome composition, we employed 16S rRNA sequencing to analyze four saliva samples, two diagnosed as OLP and 2 as NSIL. Segment of 283pb corresponding to the V3-V4 region of the 16S rRNA gene from saliva samples of all patients generated 258,820 sequences, with a total number of 194 assigned OTUs. Different values of phylogenetic diversity among groups can be observed, with a significant increase in diversity from NSIL group (3.07 ± 0.48) to OLP (4.57 ± 0.05), p < 0.05, Student t-Test (Fig. 3). Figure 4 shows the relative abundance of bacterial phyla in different groups. Eight different phyla were identified, with a higher average abundance of Firmicutes in the OLP group and of Proteobacteria in the NSIL group. In addition, the phylum Actinobacteria was present in greater abundance in the OLP group compared to NSIL. Also, 142 different species were identified in NSIL while OLP had 164 identified species in at least one sample; in the NSIL group there was a greater abundance of *Haemophilus parahaemolyticus*, *Haemophilus parainfluenza*, *Neisseria oralis*, *Streptococcus oralis*, *Streptococcus salivarius* and *Streptococcus sanguinis* species, and in the OLP group greater abundance of the species *Campylobacter rectus*, *Fusobacterium nucleatum* and *Neisseria mucosa*. (Supplementary data). We than constructed random forest classifiers to predict the disease state based on microbiome composition. Discrimination of OLP and NSIL groups based on the number of OTUs has shown that all OLP individuals were correctly classified, as demonstrated by the confusion matrix in Fig. 5A. OTUs which improved prediction of NSIL to OLP were determined in decreasing order of accuracy (Fig. 5B). The species *Streptococcus salivarius*, *Streptococcus gordonii*, *Streptococcus australis*, *Peptonaerobacter stomatis* and *Streptococcus pneumoniae* contributed most to model accuracy in classification of NSIL patients.

## Discussion

In this study we found that OLP lesions in comparison to control lesions are characterized in the lamina propria by a more severe dense lymphocytic inflammatory infiltrate in which CD3+ cells predominate. In the epithelial layer, OLP lesions have a higher frequency of apoptotic cells and of CD4+ cells in comparison NSIL samples. Regarding the expression of cytokines in the lesions we found higher expression of IFN-γ and IL-33 in OLP whereas no differences were found in IL-17 expression levels in comparison to control samples.

The characterization of the inflammatory infiltrate in OLP lesions is an important aspect and should be considered for disease diagnosis. We could observe in our OLP samples that the nature and composition of the inflammatory infiltrate corroborate other studies, which also report the presence of moderate to intense inflammatory infiltrate formed by CD4 and CD8 lymphocytes in OLP lesions^5,6,11,26^.

We found in OLP lesions a band of dense lymphomononuclear infiltrate along the interface between epithelium and lamina propria. Among the histopathological features analyzed in our study, we observed a significant association between the diagnosis of OLP and the presence of apoptotic cells. Because keratinocytes in the basal layer of the epithelium are the main target of destruction in these lesions, our results corroborate previous published findings^2,5,6,8,9^. In fact, the presence of apoptotic cells in OLP lesions is considered an important feature and adds up as a criterion to the diagnosis^5,7^.

The oral microbiota can be correlated with this type of lesions, and we were able to demonstrate that the presence of *Campylobacter rectus*, *Fusobacterium nucleatum* and *Neisseria mucosa* was associated with OLP. Furthermore, different values of phylogenetic diversity among groups can be observed, with a significant increase in diversity for OLP samples. The concept of a high microbial diversity associated with health seems to be true solely for the intestinal microbiome, but not for the microbiota of other sites of the human body^27–29^. Under conditions of bacterial vaginosis, for example, we can clearly see an increase in microbial diversity^30^. Specifically in the oral cavity, an increase in the diversity of the oral microbiota has been reported by several authors in conditions of periodontal disease^31–33^. In the recently proposed dysbiosis model of periodontitis, the disease is caused ultimately by a dysbiotic change in the biofilm microbiota that triggers an inflammatory host response^34^. Whether a similar process is occurring in OLP should be further investigated in larger sample sizes.

Regarding the cytokine genic expression analysis, our results showing high levels of IFN-γ expression in OLP samples corroborate the findings by other authors that imply a Th1 response as predominant in this disease^5,7,26,35^.

Our data also showed significant expression of IL-33 in OLP lesions in comparison to NSIL samples. To this date there is only one study on the genic expression of IL-33 in lesions from the oral cavity. Javvadi *et al*. recently investigated IL-33 and IL-35 in OLP samples and control samples from nonspecific inflammation lesions^15^. The results of immunohistochemistry showed positive staining for IL-33 and IL-35 cells in both groups evaluated, with higher number of IL33 cells found in OLP lesions. Regarding gene expression, different from our results, the authors found no difference in IL-33 levels between the two groups evaluated in their study.

The role of IL-33 in the OLP lesions physiopathology is still not quite clear. The presence of IL-33 has been reported in the lesions and in the serum of patients in a variety of allergic and autoimmune diseases^36,37^. The cytokine IL-33 is constitutively expressed by endothelial cells, keratinocytes and epithelial cells from tissues exposed to the environment and can be released in response to several types of injury to rapidly activate the innate immune response besides activating also a Th2 or supporting a Th1 type response^38^. The expression of IL-33 by human skin keratinocytes is also under regulation of IFN-γ, a Th1 signature cytokine, in a dose and time-dependent manner, as shown by Meephansan *et al*.^39^. It is worth stressing that our findings of concomitant expression of high levels of both IFN- γ and IL-33 in OLP lesions find support in those results.

Lastly, about IL-17 expression levels in OLP in comparison to NSIL samples, our results showed variable or absent expression in both types of lesions. Our results corroborate those by Javvadi *et al*. 2016 who found no significant difference in IL-17 cytokine gene expression between the OLP group and a control group^8^. However other authors found increased expression of this cytokine in lesions from oral and cutaneous lichen planus when compared to a healthy control group^40^.

Other two studies report the elevated IL-17 expression levels in OLP lesions of a particular type, namely erosive type lesions. High serum levels of this cytokine and elevated IL-17 expression were found in patients with the erosive type of OLP lesions but not in lesions of the reticular type^12,41^. In this same direction, Piccinni *et al*. 2014, analyzed clones of CD4 lymphocytes derived from OLP samples, indicating that Th17 cells are related to the more aggressive erosive oral lichen planus type of lesions^42^.

Regarding the salivary protein cytokine analysis, our results showed higher levels of IFN-γ protein expression in OLP samples, which corroborates to our genic expression data. The only OLP sample that showed lower levels of IFN-γ protein was the same sample that presented higher levels of IL-17 genic expression, and according to literature the Th17 differentiation is represented by high levels of IL-17 and only occurs in absence or low levels of IFN-γ^43^. In addition, this sample histopathology displayed suprabasal bubbles, basement layer liquefaction, and the highest levels of epithelial and conjunctive tissue infiltration with CD4/CD8 lymphocytes, suggesting a correlation between IL-17 genic expression and more aggressive lesions. Our results also corroborate the salivary levels of IFN-γ found in a study conducted by Malekzadeh *et al*.^44^. Their study aimed to investigate the correlation between salivary levels of IFN-γ and IL-4 with OLP (data from patients with reticular and erosive lesions). Their results showed higher salivary IFN-γ levels in reticular OLP patients compared to the control group, indicating significant differences between groups, and they also demonstrated that salivary IFN-γ/IL-4 ratio was significantly increased compared to control group, suggesting that Th1 cells profile is predominant over Th2 cells in OLP.

A previous study showed that OLP patient displayed low-level IFN-γ but high-level IL-4 expression, with a lower ratio of salivary IFN-γ/IL-4 compared to healthy controls. Regarding the lesions subtypes, salivary IL-4 level in erythematous/ulcerative group was significantly higher than that in reticular group. So, these data indicated a probable implication of Th2 profile in OLP, more predominant in erythematous/ulcerative group^45^. In 2013, this same group of researchers, investigated expression profile of IFN-γ and IL-4 in serum and saliva and their association. Data demonstrated that OLP patient showed low-level of IFN-γ but high-level IL-4 expression profile in serum and saliva^46^, do not corroborating our findings which demonstrate higher IFN-γ expression in OLP samples compared to control group.

Recently, a study conducted by Wei *et al*. corroborates our findings regarding IFN-γ data^47^. In this study they measured Th1/Th2 - related cytokine expression in saliva from patients with oral lichen planus (OLP) compared with healthy controls. This study showed a significantly higher expression of IFN-γ, IFN-γ/IL-4, IL-6 and IL-10 in saliva of OLP group compared to healthy controls group, which may indicate higher levels of both Th1/Th2 cytokine profile in OLP^47^. The use of saliva for diagnostic means has advantages, including ease of handling and performing, and could be a promising cost-effective tool for supporting OLP diagnosis.

Consequently, in conclusion, oral lichen planus is a mucocutaneous disease mediated by a Th1 predominant response, where IL-33 may be important and related to the maintenance of the OLP inflammatory process. Th17 in this study displayed variable expression and could not be related to the disease. Microbiota seems to be an interesting toll for supporting OLP diagnosis, and also may play a role in the etiology of the disease deserving to be further investigated. Considering that establishing the diagnosis of OLP is often a challenge to professionals, it is worth to stress that an accurate and reliable diagnosis would be better guided by clinical, microbiological and histopathological criteria, independent of the lesion subtype.

## Supplementary information

Appendix Table IV
