# Supplementary material for: Cytokines Levels and Salivary Microbiome Play A Potential Role in Oral Lichen Planus Diagnosis
Source: Sci Rep. 2019 Dec 2;9:18137. doi: 10.1038/s41598-019-54615-y (PMC6889227; doi:10.1038/s41598-019-54615-y)
Supplement: Supplementary file 1 — Appendix Table IV [file 41598_2019_54615_MOESM1_ESM.docx]

**CYTOKINES LEVELS AND SALIVARY MICROBIOME PLAY A POTENTIAL ROLE IN ORAL LICHEN PLANUS DIAGNOSIS**

Maria Fernanda Marques Silva de Carvalho^1^, Denise Cavalieri^2^, Sabrina do Nascimento^1^, Talita Gomes Baeta Lourenço^3^, Danielle Viana Ribeiro Ramos^4^, Denise da Cunha Pasqualin^5^, Leandro Aurélio Liporoni Martins^5^, Fernanda Agostini Rocha^1^, Débora Heller^1,6^, Luciana Marti^1^.

1. Hospital Israelita Albert Einstein, Experimental Research - São Paulo, Brazil
2. Secretaria Municipal de Saúde de São Paulo, Centro de Especialidades Odontológicas/Estomatologia – Unidade Alta da Boa Vista - São Paulo, Brazil
3. Universidade Federal do Rio de Janeiro, Oral Microbiology Laboratory - Rio de Janeiro, Brazil.
4. Hospital Israelita Albert Einstein - Instituto de Responsabilidade Social – Programas Governamentais - São Paulo, Brazil
5. Hospital Israelita Albert Einstein - Laboratório de Anatomia Patológica - São Paulo, Brazil
6. Universidade Cruzeiro do Sul - College of Dentistry - São Paulo, Brazil.

**SUPPLENTARY INFORMATION**

**Appendix Table IV.** Heat map of bacteria’s present in saliva according to NSIL or OLP samples.

| **Species** | **NSIL (1)** | **NSIL (2)** | **OLP (1)** | **OLP (2)** |
| --- | --- | --- | --- | --- |
| Abiotrophia defectiva | 461 | 0 | 18 | 23 |
| Acidithiobacillus thiooxidans | 0 | 0 | 819 | 0 |
| Actinomyces genomosp. C1 | 2 | 0 | 0 | 0 |
| Actinomyces gerencseriae | 1 | 0 | 3 | 0 |
| Actinomyces graevenitzii | 0 | 17 | 1 | 0 |
| Actinomyces johnsonii | 2 | 0 | 15 | 0 |
| Actinomyces lingnae | 0 | 1 | 2 | 0 |
| Actinomyces massiliensis | 30 | 0 | 26 | 18 |
| Actinomyces naeslundii | 200 | 0 | 23 | 151 |
| Actinomyces odontolyticus | 2 | 4 | 2 | 8 |
| Actinomyces oris | 113 | 0 | 18 | 0 |
| Actinomyces sp. | 0 | 8 | 4 | 1 |
| Actinomyces viscosus | 170 | 0 | 88 | 50 |
| Aggregatibacter aphrophilus | 100 | 0 | 547 | 11 |
| Aggregatibacter segnis | 165 | 0 | 50 | 20 |
| Alloprevotella rava | 5 | 1 | 0 | 3 |
| Alloprevotella tannerae | 0 | 0 | 3 | 72 |
| Alloscardovia omnicolens | 3 | 0 | 0 | 0 |
| Anaeroglobus geminatus | 0 | 0 | 0 | 32 |
| Atopobium parvulum | 1 | 6 | 5 | 21 |
| Atopobium rimae | 0 | 0 | 0 | 5 |
| Bifidobacterium dentium | 2 | 0 | 0 | 0 |
| Bradyrhizobium genosp. SA-1 | 1 | 0 | 1 | 5 |
| Brochothrix thermosphacta | 3 | 0 | 0 | 0 |
| Bulleidia extructa | 0 | 0 | 0 | 21 |
| Burkholderia cepacia | 0 | 0 | 1 | 0 |
| Burkholderia fungorum | 0 | 0 | 0 | 6 |
| Campylobacter concisus | 302 | 63 | 131 | 280 |
| Campylobacter curvus | 0 | 0 | 30 | 7 |
| Campylobacter gracilis | 39 | 0 | 41 | 36 |
| Campylobacter rectus | 241 | 1 | 4392 | 165 |
| Campylobacter showae | 1 | 0 | 142 | 1 |
| Candidatus Prevotella conceptionensis | 11 | 0 | 8 | 10 |
| Capnocytophaga gingivalis | 4 | 0 | 162 | 0 |
| Capnocytophaga granulosa | 0 | 0 | 18 | 2 |
| Capnocytophaga haemolytica | 14 | 0 | 0 | 0 |
| Capnocytophaga leadbetteri | 0 | 0 | 71 | 3 |
| Capnocytophaga ochracea | 8 | 0 | 5 | 7 |
| Capnocytophaga sp. | 0 | 0 | 28 | 4 |
| Capnocytophaga sputigena | 11 | 0 | 0 | 2 |
| Cardiobacterium hominis | 6 | 0 | 5 | 0 |
| Cardiobacterium valvarum | 16 | 0 | 58 | 2 |
| Catonella morbi | 15 | 3 | 5 | 13 |
| Centipeda periodontii | 4 | 0 | 0 | 6 |
| Cloacibacterium normanense | 0 | 1 | 0 | 0 |
| Corynebacterium durum | 18 | 0 | 37 | 2 |
| Corynebacterium kroppenstedtii | 2 | 0 | 0 | 0 |
| Corynebacterium matruchotii | 71 | 0 | 172 | 6 |
| Corynebacterium tuberculostearicum | 5 | 1 | 0 | 0 |
| Desulfomicrobium orale | 0 | 0 | 7 | 0 |
| Dialister invisus | 12 | 0 | 14 | 15 |
| Dialister pneumosintes | 4 | 0 | 0 | 77 |
| Eikenella corrodens | 12 | 0 | 50 | 29 |
| Enterobacteriaceae sp. | 15 | 5 | 8 | 7 |
| Escherichia coli | 0 | 0 | 0 | 8 |
| Eubacterium brachy | 0 | 0 | 6 | 277 |
| Eubacterium saphenum | 0 | 0 | 2 | 0 |
| Filifactor alocis | 23 | 0 | 109 | 0 |
| Fretibacterium fastidiosum | 0 | 0 | 0 | 66 |
| Fusobacterium nucleatum | 635 | 0 | 2687 | 1420 |
| Fusobacterium periodonticum | 20 | 162 | 975 | 43 |
| Fusobacterium simiae | 0 | 0 | 4 | 3 |
| Granulicatella adiacens | 52 | 52 | 76 | 104 |
| Granulicatella elegans | 415 | 377 | 324 | 160 |
| Haemophilus genomosp. P3 oral clone MB3_C38 | 0 | 0 | 0 | 42 |
| Haemophilus haemolyticus | 371 | 265 | 82 | 132 |
| Haemophilus influenzae | 4 | 7 | 4 | 0 |
| Haemophilus parahaemolyticus | 0 | 5537 | 2169 | 8 |
| Haemophilus parainfluenzae | 6202 | 4673 | 3820 | 34 |
| Haemophilus pittmaniae | 1 | 142 | 165 | 0 |
| Haemophilus sputorum | 6 | 34 | 634 | 0 |
| Janibacter limosus | 0 | 0 | 1 | 0 |
| Johnsonella ignava | 11 | 0 | 0 | 2 |
| Kingella denitrificans | 1 | 0 | 0 | 2 |
| Kingella oralis | 42 | 0 | 10 | 120 |
| Kocuria palustris | 2 | 0 | 0 | 0 |
| Lachnoanaerobaculum cf. saburreum oral strain C27KA | 5 | 15 | 329 | 16 |
| Lachnoanaerobaculum orale | 3 | 4 | 0 | 12 |
| Lachnoanaerobaculum saburreum | 56 | 0 | 3 | 3 |
| Lachnoanaerobaculum umeaense | 0 | 0 | 14 | 0 |
| Lactococcus lactis | 17 | 0 | 0 | 0 |
| Lactococcus piscium | 3 | 0 | 0 | 0 |
| Lautropia mirabilis | 41 | 0 | 80 | 48 |
| Leptotrichia buccalis | 91 | 0 | 702 | 2 |
| Leptotrichia goodfellowii | 4 | 0 | 6 | 37 |
| Leptotrichia hofstadii | 119 | 0 | 106 | 0 |
| Leptotrichia hongkongensis | 114 | 0 | 29 | 5 |
| Leptotrichia shahii | 34 | 0 | 0 | 1 |
| Leptotrichia trevisanii | 8 | 0 | 0 | 0 |
| Leptotrichia wadei | 8 | 0 | 0 | 42 |
| Megasphaera genomosp. C1 | 0 | 1 | 0 | 0 |
| Megasphaera micronuciformis | 0 | 9 | 0 | 1 |
| Mogibacterium neglectum | 2 | 0 | 1 | 7 |
| Mogibacterium timidum | 0 | 0 | 0 | 10 |
| Mycoplasma faucium | 1 | 0 | 0 | 1 |
| Mycoplasma salivarium | 1 | 0 | 1 | 0 |
| Neisseria bacilliformis | 0 | 0 | 45 | 8 |
| Neisseria elongata | 27 | 0 | 141 | 12 |
| Neisseria flavescens | 13 | 10 | 0 | 25 |
| Neisseria mucosa | 31 | 0 | 1353 | 103 |
| Neisseria oralis | 1407 | 0 | 0 | 0 |
| Neisseria perflava | 2 | 0 | 1 | 28 |
| Neisseria pharyngis | 0 | 10 | 0 | 0 |
| Neisseria subflava | 43 | 0 | 0 | 10 |
| Oribacterium sinus | 2 | 16 | 16 | 3 |
| Pantoea agglomerans | 1 | 0 | 0 | 0 |
| Paracoccus sphaerophysae | 0 | 0 | 3 | 0 |
| Paraprevotella clara | 1 | 0 | 47 | 0 |
| Parvimonas micra | 31 | 0 | 42 | 420 |
| Peptoniphilus lacrimalis | 0 | 0 | 0 | 5 |
| Peptostreptococcus stomatis | 0 | 0 | 34 | 1 |
| Porphyromonas catoniae | 4 | 0 | 0 | 5 |
| Porphyromonas endodontalis | 13 | 0 | 33 | 134 |
| Porphyromonas gingivalis | 0 | 0 | 684 | 0 |
| Prevotella bivia | 6 | 0 | 0 | 0 |
| Prevotella denticola | 1 | 0 | 0 | 27 |
| Prevotella fusca | 0 | 0 | 0 | 2 |
| Prevotella histicola | 1 | 29 | 4 | 9 |
| Prevotella loescheii | 1 | 0 | 2 | 0 |
| Prevotella maculosa | 0 | 0 | 7 | 26 |
| Prevotella melaninogenica | 71 | 84 | 302 | 240 |
| Prevotella micans | 1 | 0 | 10 | 20 |
| Prevotella multisaccharivorax | 5 | 0 | 0 | 0 |
| Prevotella nanceiensis | 0 | 51 | 100 | 39 |
| Prevotella nigrescens | 2 | 0 | 0 | 6 |
| Prevotella oris | 8 | 0 | 10 | 92 |
| Prevotella oulorum | 26 | 0 | 0 | 20 |
| Prevotella pallens | 5 | 3 | 8 | 2 |
| Prevotella pleuritidis | 0 | 0 | 0 | 48 |
| Prevotella saccharolytica | 0 | 0 | 34 | 0 |
| Prevotella salivae | 38 | 24 | 4 | 227 |
| Prevotella shahii | 0 | 0 | 1 | 10 |
| Prevotella veroralis | 5 | 0 | 0 | 34 |
| Pseudomonas fluorescens | 0 | 0 | 0 | 3 |
| Pseudomonas putida | 0 | 0 | 0 | 7 |
| Psychrobacter aquimaris | 1 | 0 | 0 | 0 |
| Rothia aeria | 332 | 0 | 189 | 639 |
| Rothia dentocariosa | 67 | 2 | 229 | 159 |
| Rothia mucilaginosa | 31 | 450 | 450 | 1211 |
| Rubrobacter xylanophilus | 2 | 0 | 0 | 0 |
| Selenomonas artemidis | 5 | 0 | 82 | 1 |
| Selenomonas dianae | 0 | 0 | 0 | 21 |
| Selenomonas genomosp. C2 | 0 | 0 | 1 | 0 |
| Selenomonas genomosp. P6 oral clone MB3_C41 | 18 | 0 | 28 | 0 |
| Selenomonas genomosp. P8 oral clone MB5_P06 | 1 | 0 | 0 | 1 |
| Selenomonas infelix | 3 | 0 | 30 | 26 |
| Selenomonas noxia | 276 | 0 | 796 | 23 |
| Selenomonas sputigena | 5 | 0 | 13 | 56 |
| Shuttleworthia satelles | 1 | 0 | 0 | 0 |
| Solobacterium moorei | 2 | 0 | 2 | 5 |
| Sphingomonas leidyi | 0 | 0 | 0 | 1 |
| Staphylococcus capitis | 0 | 0 | 1 | 0 |
| Staphylococcus caprae | 0 | 0 | 0 | 1 |
| Staphylococcus epidermidis | 4 | 2 | 3 | 0 |
| Staphylococcus hominis | 0 | 0 | 0 | 1 |
| Stomatobaculum longum | 1 | 8 | 2 | 0 |
| Streptococcus agalactiae | 0 | 0 | 0 | 3 |
| Streptococcus anginosus | 4 | 1 | 1 | 4 |
| Streptococcus australis | 14 | 432 | 299 | 63 |
| Streptococcus constellatus | 0 | 0 | 1 | 27 |
| Streptococcus cristatus | 0 | 5 | 0 | 0 |
| Streptococcus gordonii | 137 | 24 | 12 | 15 |
| Streptococcus intermedius | 6 | 0 | 11 | 81 |
| Streptococcus mitis | 333 | 105 | 41 | 331 |
| Streptococcus mutans | 7 | 0 | 0 | 0 |
| Streptococcus oralis | 3369 | 8749 | 2338 | 1378 |
| Streptococcus parasanguinis | 4 | 151 | 10 | 59 |
| Streptococcus pneumoniae | 13 | 65 | 7 | 6 |
| Streptococcus salivarius | 2294 | 789 | 70 | 50 |
| Streptococcus sanguinis | 1580 | 11 | 114 | 140 |
| Streptococcus sinensis | 7 | 15 | 2 | 10 |
| Streptococcus thermophilus | 4 | 3 | 2 | 0 |
| Streptococcus vestibularis | 1 | 0 | 0 | 0 |
| Tannerella forsythia | 17 | 0 | 75 | 0 |
| Terrahaemophilus aromaticivorans | 88 | 778 | 423 | 9 |
| Treponema denticola | 0 | 0 | 3 | 0 |
| Treponema lecithinolyticum | 0 | 0 | 16 | 0 |
| Treponema medium | 0 | 0 | 0 | 7 |
| Treponema parvum | 0 | 0 | 2 | 1 |
| Treponema socranskii | 0 | 0 | 2 | 16 |
| Veillonella atypica | 0 | 21 | 0 | 3 |
| Veillonella denticariosi | 128 | 4 | 32 | 0 |
| Veillonella dispar | 8 | 294 | 0 | 28 |
| Veillonella genomosp. P1 oral clone MB5_P17 | 19 | 73 | 2 | 2 |
| Veillonella parvula | 230 | 68 | 150 | 56 |
| Veillonella rogosae | 0 | 118 | 35 | 39 |
| Veillonella tobetsuensis | 6 | 4 | 25 | 3 |
| [Eubacterium] yurii | 40 | 0 | 6 | 0 |
